# Development, Characterization and Incorporation of Alginate-Plant Protein Covered Liposomes Containing Ground Ivy (*Glechoma hederacea* L.) Extract into Candies

**DOI:** 10.3390/foods11121816

**Published:** 2022-06-20

**Authors:** Danijela Šeremet, Martina Štefančić, Predrag Petrović, Sunčica Kuzmić, Shefkije Doroci, Ana Mandura Jarić, Aleksandra Vojvodić Cebin, Rada Pjanović, Draženka Komes

**Affiliations:** 1 Department of Food Engineering, Faculty of Food Technology and Biotechnology, University of Zagreb, Pierotti St 6, 10 000 Zagreb, Croatia; dseremet@pbf.hr (D.Š.); martinastefancic87@gmail.com (M.Š.); sdoroci@pbf.hr (S.D.); amandura@pbf.hr (A.M.J.); avojvodic@pbf.hr (A.V.C.); 2 Department of Chemical Engineering, Faculty of Technology and Metallurgy, University of Belgrade, Karnegijeva 4, 11 000 Belgrade, Serbia; ppetrovic@tmf.bg.ac.rs (P.P.); rada@tmf.bg.ac.rs (R.P.); 3 Forensic Science Centre “Ivan Vučetić” Zagreb, Forensic Science Office, University of Zagreb, Ilica 335, 10 000 Zagreb, Croatia; skuzmic@mup.hr

**Keywords:** encapsulation, ground ivy, liposomes, plant proteins, polyphenols

## Abstract

Ground ivy (*Glechoma hederacea* L.) has been known as a medicinal plant in folk medicine for generations and, as a member of the *Lamiaceae* family, is characterized with a high content of rosmarinic acid. The aim of the present study was to formulate delivery systems containing bioactive compounds from ground ivy in encapsulated form and incorporated into candies. Liposomes were examined as the encapsulation systems that were additionally coated with an alginate–plant protein gel to reduce leakage of the incorporated material. Bioactive characterization of the ground ivy extract showed a high content of total phenolics (1186.20 mg GAE/L) and rosmarinic acid (46.04 mg/L). The formulation of liposomes with the high encapsulation efficiency of rosmarinic acid (97.64%), with at least a double bilayer and with polydisperse particle size distribution was achieved. Alginate microparticles reinforced with rice proteins provided the highest encapsulation efficiency for rosmarinic acid (78.16%) and were therefore used for the successful coating of liposomes, as confirmed by FT-IR analysis. Coating liposomes with alginate–rice protein gel provided prolonged controlled release of rosmarinic acid during simulated gastro-intestinal digestion, and the same was noted when they were incorporated into candies.

## 1. Introduction

*Lamiaceae* is one of the largest plant families worldwide, consisting of more than 200 genera and 7000 species. Many of them are used in the cuisine, pharmaceutical and cosmetic industries due to their spicy and aromatic properties. Some of them are also appreciated in traditional medicine for the treatment of different diseases (asthma, depression, respiratory and Alzheimer’s diseases, etc.), due to the biological activities arising from the presence of phenolic compounds, especially rosmarinic acid [1]. As a member of the *Lamiaceae* family, ground ivy (*Glechoma hederacea* L.) shares a common characteristic of a high content of rosmarinic acid [2,3]. Rosmarinic acid was first isolated in 1958 by Italian scientists Scarpati and Oriente from the plant *Rosmarinus officinalis*, after which it was named. By its structure, rosmarinic acid is an ester of caffeic acid and 3,4-dihydroxyphenyl lactic acid [4]. Rosmarinic acid possesses antidiabetic, antimicrobial, cardioprotective, antioxidant, hepatoprotective, anti-allergic, anti-inflammatory activity, etc., [5] and as such has great potential as a natural ingredient for the fortification of food products. Despite its numerous benefits, the incorporation of rosmarinic acid into food products is limited due to its chemical instability, low water solubility and discoloration [6]; as one of the solutions, encapsulation has been suggested.

Encapsulation can be defined as an entrapping technique for core material into a matrix of inert wall material. It meets the requirements of the food industry for providing protection from the environmental stressors and enhancing the shelf life of unstable bioactive compounds and their delivery at the target site at a controlled rate. Recent trends are also focused on their reduced toxicity, reduced cost and enhanced bioavailability [7]. Only wall materials that are certified as “generally recognized as safe” (GRAS) can be used in food-applications lipids (waxes, glycerides, phospholipids, etc.), proteins (corn gluten, caseins, gelatin, etc.) and carbohydrate polymers (gum arabic, cellulose, starch, alginate, etc.) whether they are of plant, animal, microbial or marine origin [7]. Alginate is one of the most studied wall materials, mainly used in spray drying, extrusion and emulsification/gelation techniques [8]. In order to increase the encapsulation efficiency or reduce the release of the core material, one of the proposed methods is to blend the alginate with other biopolymers, such as proteins. Complexes between polysaccharides and proteins form spontaneously in an aqueous solution due to the electrostatic interactions between groups with opposite charges [9,10]. Plant proteins are increasingly used in the human diet as alternatives to animal-originated proteins and can satisfy nutritional needs in terms of required essential amino acids [11]. From a technological aspect, their role in providing a higher retention of core materials when incorporated into microparticles, was proven by Bušić et al. [12] on the example of zein–alginate microparticles, and by Silverio et al. [13] with the electrostatic adsorption of concentrated soy protein on alginate microparticles. Liposomes, on the other hand, are lipid vesicles composed of one or more lipid bilayers characterized by a spherical structure and amphiphilic character that makes them suitable for the encapsulation of both hydrophilic and hydrophobic compounds [14]. Although liposomes were described as drug delivery system decades ago, more precisely in 1971, by British scientists Gregoriadis and Ryman, only a few liposomal drugs are today available on the market [14]. In the food industry, liposomes are mainly used to encapsulate antimicrobials in dairy and meat products and vitamins in beverages [15], while the approach of using them as delivery systems for natural antioxidants in food to improve their bioavailability is less represented. 

Candies are among the most popular confectionery products consumed by all generations, so they are a good choice for the delivery of bioactive compounds for human health to a large number of people [16]. One of the main reasons for the consumption of candies is sweet and pleasant taste that triggers positive emotions, so the present study aimed to formulate candies that will satisfy sensory requirements and simultaneously provide a source of natural antioxidants. Dynamic changes on the global candy market are affected by increased health consciousness, emphasized consumer’s demands for organic, vegan and “clean-labelled” food products with unconventional or exotic flavors [17]. Considering mentioned trends, in the present study, ground ivy (*Glechoma hederacea* L.) was chosen as a plant material for the extraction of phenolic compounds that will be incorporated in encapsulated form into candies. Liposomes were examined as the encapsulation system and since they are prone to leakage and losing the encapsulated content over time [18], the loaded liposomes were additionally coated with alginate reinforced with plant proteins. The encapsulation efficiency, physical parameters and simulated gastro-intestinal digestion of both liposomes and microparticles were examined.

## 2. Materials and Methods

### 2.1. Materials

Ground ivy was harvested in April 2020 on the area of Bilogora (Bjelovar, Croatia). The identification and harvesting were performed by botanists with the previous experience in collecting plant materials. The sample of harvested ground ivy was stored in Flora Croatica Database (Department of Botany, Faculty of science, University of Zagreb) under number 71767. Aerial parts were separated from roots and air-dried at room temperature for 3 days to the content of dry matter >90% and afterwards ground and sieved to obtain a fraction (<450 µm) for further applications. Rice (fat content: 4.5%, carbohydrates content: 2.9% and protein content: 83.0%), peanut (fat content: 13.3%, carbohydrates content: 26.6% and protein content: 53.3%) and pumpkin (fat content: 10.8%, carbohydrates content: 17.3% and protein content: 51.4%) protein powders were obtained from Nutrigold (Zagreb, Croatia), xylitol and agar-agar from bio&bio (Zagreb, Croatia).

### 2.2. Chemicals

Folin–Ciocalteu’s reagent, calcium chloride and hydrochloric acid were supplied from Kemika (Zagreb, Croatia). Gallic (>97%) and rosmarinic (97%) acid, (S)-6-methoxy-2,5,7,8-tetramethylchromane-2-carboxylic acid (Trolox), 2,2-diphenyl-1-picrylhydrazyl (DPPH), 2,2′-azino-bis(3-ethylbenzothiazoline-6-sulfonic acid) diammonium salt (ABTS), 3-(2-pyridyl)-5,6-diphenyl-1,2,4-triazine-p,p′-disulfonic acid monosodium salt hydrate (ferrozine), linoleic acid, alginic acid sodium salt from brown algae (sodium alginate, low viscosity), pepsin from porcine gastric mucosa, bile bovine and pancreatin from porcine pancreas were purchased from Sigma-Aldrich (St. Louis, MO, USA). Ethanol, formic acid, potassium chloride and sodium chloride were purchased from Carlo Erba (Emmendingen, Germany),methanol from Panreac (Barcelona, Spain), chloroform and iron (II) sulfate heptahydrate from Gram-mol d.o.o. (Zagreb, Croatia), acetonitrile from Fisher Scientific (Waltham, MA, USA) and sodium citrate from T.T.T. (Sveta Nedelja, Croatia). Tween^®^ 80 was supplied by Acros Organics (Waltham, MA, USA), potassium peroxodisulfate and β-carotene by Fluka (Buchs, Switzerland). Potassium dihydrogen phosphate, sodium hydrogen carbonate, magnesium chloride hexahydrate, ammonium carbonate and sodium hydroxide were purchased from Lach-Ner (Neratovice, Czech Republic). Phospholipon^®^ 90G (purified phosphatidylcholine from soybean lecithin; P90G) was supplied by Lipoid GmbH (Ludwigshafen, Germany). All chemicals used for experimental procedures were of analytical or HPLC grade.

### 2.3. Methods

#### 2.3.1. Preparation of Ground Ivy Extract

The extraction of 1 g of ground ivy sample with 25 mL of distilled water was carried out in water bath Inko VKZ ERN (Inkolab d.o.o., Zagreb, Croatia) for 10 min at a temperature of 100 °C. Upon the complementation of extraction, centrifugation (Thermo Scientific SL8/8R centrifuge, Waltham, MA, USA; 9500 rpm, 20 min) and filtration (Whatman^®^ filter papers 4) were performed. After filtration, the extract was immediately subjected to the analyses. 

#### 2.3.2. Bioactive Characterization of the Extract

The obtained extract was characterized using spectrophotometric (Genesys 10S UV-VIS Spectrophotometer, Thermo Fisher Scientific, Waltham, MA, USA) and chromatographic methods described below. All measurements were performed in triplicate. 

##### Determination of Total Phenolic Content (TPC)

Determination of TPC was conducted using Folin–Ciocalteu’s reagent [19] and gallic acid for the construction of calibration curve. Reaction mixture consisted of distilled water (3.75 mL), diluted extract (50 µL), Folin–Ciocalteu’s reagent diluted with water in ratio 1:2 (250 µL) and 20% (*w*/*v*) Na_2_CO_3_ solution (750 µL). The absorbance of the reaction mixture was measured at 765 nm after 2 h.

##### DPPH Radical Scavenging Activity

The determination of the antioxidant capacity by applying DPPH radical cation decolorization assay was performed with Trolox as a standard for the calibration curve [20]. The reaction mixture consisted of diluted extract (100 μL) and 0.094 mM DPPH solution in methanol (3.9 mL). The absorbance was measured after 30 min at 515 nm.

##### ABTS^•+^ Decolorization Assay

Determination of antioxidant capacity by applying ABTS^•+^ radical cation decolorization assay was performed with Trolox as a standard for the calibration curve [21]. The 7 mM ABTS solution (4.912 mL) was mixed with 140 mM potassium peroxodisulfate (88 µL) in water and left to react 16 h in the dark. Prior to the analysis, the ABTS^•+^ radical solution was diluted with ethanol to an absorbance of 0.700 at 734 nm. The reaction mixture consisted of diluted extract (40 μL) and of the ABTS^•+^ radical solution (4.0 mL). The absorbance was measured at 734 nm after 6 min.

##### Inhibition of β-Carotene Bleaching

The inhibition of β-carotene bleaching was performed using the β-carotene-linoleic acid system described by Ferreira et al. [22] and was evaluated using the following formula (Equation (1)):

(1) $$\%\;inhibition=\;\frac{R_{control}-R_{sample}}{R_{control}\;}\times 100$$

where *R* corresponds to bleaching rate and was assessed as (Equation (2)) [23]:

(2) $$R=\ln\left(\frac{initial\;absorbance\;at\;420\;\mathrm{nm}}{absorbance\;at\;420\;\mathrm{nm}\;after\;2\;h}\right)/t$$

##### Metal Chelating Capacity

Metal chelating capacity was performed by the method described by Adusei et al. [24]. The reaction mixture consisted of extract (1 mL), 0.1 mM FeSO_4_ (1 mL) and 0.25 mM ferrozine (1 mL). The absorbance was measured at 562 nm after 10 min. The metal chelating capacity was evaluated as (Equation (3)):

(3) $$\%\;chelation=\left(1-\frac{A_{sample}-A_{blank}}{A_{control}}\right)\times 100$$

where blank contained no ferrozine, while control no extract [25].

##### Determination of Rosmarinic Acid

The content of rosmarinic acid was determined on an Agilent Series 1200 chromatographic system (Agilent Technologies, Santa Clara, CA, USA) using a Zorbax Extend C18 (4.6 × 250 mm, 5 μm i.d.) chromatographic column (Agilent Technologies, Santa Clara, CA, USA) and coupled with a Photodiode Array Detector (PAD) (Agilent Technologies, Santa Clara, CA, USA) (Table S1, Figure S1). The gradient elution was performed with a two-component mobile phase consisting of 1% (*v*/*v*) formic acid solution in water and 1% (*v*/*v*) formic acid solution in acetonitrile, as described by Šeremet et al. [26].

#### 2.3.3. Formulation of Alginate–Protein Microparticles

The method for the formulation of alginate–protein microparticles was adopted from the literature [12] with some modifications. Alginate–protein mixtures were prepared by dissolving sodium alginate and plant proteins in prepared ground ivy extract to achieve their final content of 3% (*w*/*w*) and 12% (*w*/*w*) in the mixture, respectively, while in the case of pure alginate, 4% (*w*/*w*) alginate solution in the extract was prepared. The prepared alginate and alginate–protein mixtures were added dropwise (Perfusor Compact Plus, B. Braun, Germany) through a metal needle (G 20) into a hardening bath containing 12% CaCl_2_ (*w*/*v*) in the case of the alginate–protein mixtures, and 4% CaCl_2_ (*w*/*v*) for the alginate mixture. Finally, the 4 formulations of microparticles were obtained: alginate (As), alginate–peanut protein (A_PEAs), alginate–rice protein (A_RICs) and alginate–pumpkin protein (A_PUMs) microparticles.

#### 2.3.4. Determination of Encapsulation Efficiency (EE) for Microparticles

TPC, antioxidant capacity and content of rosmarinic acid immobilized in formulated alginate and alginate–protein microparticles were evaluated by dissolving microparticles in of 4% (*w*/*v*) and 12% (*w*/*v*) sodium citrate, respectively, on a magnetic stirrer until complete dissolution. The method for the determination of EE was adopted from the literature [12] with some modifications. To examine the encapsulation efficiency of polyphenols originating only from ground ivy, blank samples consisting of water instead of extract were prepared and examined. The encapsulation efficiency (%) was calculated as the ratio between TPC, antioxidant capacity and content of rosmarinic acid in the citrate solution of dissolved microparticles, and their respective content in the initial delivery solution following methods described under Section 2.3.2.

#### 2.3.5. Determination of Size and Color of Microparticles

The size of the microparticles was determined using Dino-Lite calibration plate (minimal distance = 0.2 mm). Color determination was performed using a portable spectrophotometer CM-700d (Konica Minolta, Japan). Results were expressed as CIE coordinates of L* (lightness), a* (redness/greenness) and b* (yellowness/blueness). Overall color difference (∆E) was calculated from the ∆L*, ∆a*, and ∆b* using alginate microparticles as the control (Equation (4)):

(4) $$\Delta E=\sqrt{{\Delta L}^{2}+{\Delta a}^{2}+{\Delta b}^{2}}$$

#### 2.3.6. Morphology of Microparticles

Freeze-dried microparticles (Christ, Alpha 1-2 LD plus, Osteorode, Germany) were morphologically examined using scanning electron microscopy (SEM) analysis with TESCAN Mira3 microscope (Brno, Czech Republic). Microparticles were attached to stubs using two-sided adhesive tape coated with a layer of gold and examined using an acceleration voltage of 4.0 kV. 

#### 2.3.7. Liposomal Encapsulation of the Extract

For liposomal encapsulation, the extract was concentrated through evaporation under vacuum (IKA RV8, Staufen, Germany) and subjected to freeze drying (Christ, Alpha 1-2 LD plus, Osteorode, Germany) to obtain the extract in powdery form. Liposomes were prepared following the proliposome method [27]. Briefly, 1 g of P90G was dissolved in the 1.25 mL of ethanol (96%) by heating to 60 °C for 2 min in a water bath with constant stirring. Then 50 mg of freeze-dried extract was dissolved in 2 mL of distilled water and added into the prepared mixture of P90G and ethanol. The mixture was again heated up to 60 °C for 2 min and left to cool down. Finally, 48 mL of distilled water was added dropwise (Perfusor Compact Plus, B. Braun, Germany) through a metal needle (G 23) into the mixture while stirring to allow the formation of liposomes.

#### 2.3.8. Determination of Encapsulation Efficiency (EE) for Liposomes

Liposomes were subjected to centrifugation (6000 rpm, 1 h) after which supernatants were collected and subjected to analyses, while the remaining residue of the liposomes was washed once with water, centrifuged (6000 rpm, 30 h), suspended in the 1 mL of distilled water and dissolved in a chloroform–methanol (1 mL:1 mL) mixture. The mixture was vortexed for 1 min, and the phase separation was accelerated by short centrifugation. The upper water–methanol phase was collected and subjected to analyses. To measure the encapsulation efficiency, the determination of TPC, antioxidant capacity and content of rosmarinic acid, as described previously (Section 2.3.2), were performed both in collected supernatants and water–methanol phases.

#### 2.3.9. Physical Characterization of Liposomes

The size, polydispersity index (PDI) and zeta potential of liposomes were measured using a Malvern Nano-ZS Zetasizer (Malvern, UK).

#### 2.3.10. Coating the Liposomes with Alginate-Proteins Mixture

Liposomes were coated with a mixture of alginate reinforced with plant proteins that resulted in the highest EE and satisfactory physical properties (explained in the Results and Discussion section). Liposomes were mixed at a ratio of 1:1 (*w*/*w*) with the alginate–protein mixture (containing 6% (*w*/*w*) of alginate and 24% (*w*/*w*) of plant proteins in the extract). The prepared alginate–protein–liposome mixture was added dropwise (Perfusor Compact Plus, B. Braun, Germany) through a metal needle (G 20) into a hardening bath containing 12% (*w*/*v*) CaCl_2_.

#### 2.3.11. Fourier-Transform Infrared (FT-IR) Spectroscopy

FT-IR spectra of ground ivy extract, plain and loaded liposomes, alginate–protein microparticles and alginate–protein-coated liposomes were recorded in the attenuated total reflectance (ATR) mode between 400 and 4000 cm^−1^ using a Nicolet iS10 (Thermo Scientific, Waltham, MA, USA) spectrometer. Prior to analysis, samples were freeze dried.

#### 2.3.12. Preparation of Agar-Agar Candies

Agar-agar candies were prepared under laboratory conditions. A 6% (*w*/*w*) solution of agar-agar was prepared in ground ivy extract by heating and stirring at 60 °C until complete dissolution. The extract was prepared as described in Section 2.3.1. A 70% (*w*/*w*) solution of xylitol in the extract was prepared separately and heated up to 100 °C until complete dissolution and then added to the agar-agar solution in ratio 1:1 (*w*/*w*) and mixed. Two formulations of candies were prepared: one with and one without incorporated microparticles. In the case of candy formulation with microparticles, microparticles were added in an amount equal to 10% of the candy weight. The candies were prepared by pouring the prepared mixture into silicone molds and cooling and solidifying at 4 °C for 2 h.

#### 2.3.13. Bioactive Characterization of Formulated Agar-Agar Candies

The prepared agar-agar candies were cut into small pieces. The extraction of phenolic compounds from ground candies was performed using 80% (*v*/*v*) aqueous methanolic solution at ratio sample/solvent 1:20 (*w*/*v*). The first 30 min of extraction was carried out in an ultrasonic bath (Elmasonic 2 120, Elma, Singen, Germany) with a nominal power of 200 W and a frequency of 37 kHz followed by extraction on a magnetic stirrer for 15 min at room temperature. Upon complementation of extraction, centrifugation (9500 rpm, 10 min) was performed, the supernatant was separated, and the remaining residue of candies was once more subjected to the extraction under the same parameters. Supernatants of both extractions were merged and then analyzed. Extracts were subjected to the analysis of TPC, antioxidant capacity and rosmarinic acid content, following the method described under Section 2.3.2.

#### 2.3.14. Simulated Gastro-Intestinal Digestion

Simulated gastro-intestinal digestion was performed on alginate–plant protein microparticles, liposomes, liposomes coated with alginate–plant proteins and formulated candies by evaluating the rosmarinic acid content. The formulated candies were cut into small pieces before the analysis of the simulated gastro-intestinal digestion. Simulated gastro- (SGF) and intestinal (SIF) fluids were prepared as described by Minekus et al. [28]. SGF (pH = 3) contained pepsin, while SIF (pH = 7) contained pancreatin and bile bovine. During the simulated digestion, the temperature of SGF and SIF was held at 37 °C. Aliquots of the sample, taken at certain points of time, were transferred to a glass screw-capped tube, and acidified with 4 M HCl solution to reach 0.2 M concentration. The 5 M NaCl solution was added to improve phase separation in the following steps. Rosmarinic acid was then extracted by liquid–liquid extraction with ethyl acetate (1:1, *v*/*v*) by mixing on vortex for 1 min. Liquid–liquid extraction was repeated two times more. The collected organic phase was evaporated under a vacuum (IKA RV8, Staufen, Germany) to dryness. The dry residue was dissolved in 80% (*v*/*v*) methanol and subjected to HPLC analysis. Simulated gastro-intestinal digestion for each sample was performed in duplicate.

#### 2.3.15. Statistical Analysis 

One-way ANOVA and Tukey’s post hoc tests were performed in the SPSS Statistics 17.0 software with a significance level of α = 0.05. Single-factor ANOVA was performed in Microsoft Excel (Microsoft, Redmond, Washington, SAD) with a significance level of α = 0.05.

## 3. Results and Discussion

### 3.1. Bioactive Characterization of Ground Ivy Extract

The bioactive characterization of ground ivy extract included the determination of TPC, antioxidant capacity and content of rosmarinic acid. The results are presented in Table 1.

According to the obtained values of TPC (1186.20 mg GAE/L) and content of rosmarinic acid (46.04 mg/L), ground ivy can be considered a phenolic-rich plant. Additionally, its potent antioxidant properties are proven by the good scavenging activity against DPPH and ABTS (3.33 and 4.05 mmol TroloxE/L, respectively). Further, ground ivy extract exhibited ferrous ion chelation activity (74.35%), as well as relatively strong β-carotene bleaching activity (57.37%). A conclusion can be made that ground ivy can serve as a plant material for the fortification of food products with natural antioxidants. 

### 3.2. Encapsulation Efficiency (EE) of Microparticles

Microparticles with alginate (As) and in combination with rice proteins (A_RICs), pumpkin proteins (A_PUMs) and peanut proteins (A_PEAs) were formulated and the results of their EE are presented in Table 2.

Alginate by itself provided relatively good EE of all examined bioactive parameters, whereas the incorporation of pumpkin and peanut proteins did not result in a statistically significant increase (*p* > 0.05) in EE for rosmarinic acid. The A_RICs were characterized with the highest EE for TPC (84.06%), antioxidant capacity (79.09 and 81.10%) and rosmarinic acid (78.16%). The enhancement of EE by the incorporation of rice proteins can be explained by the non-covalent hydrophobic interactions between polyphenols and proteins. These interactions can be subsequently stabilized by hydrogen bonding since the phenolic group can serve as a hydrogen donor that forms strong hydrogen bonds with the carboxyl group of proteins [29,30]. It is likely that the incorporation of rice proteins into the alginate gel resulted in the formation of a denser structure with cohesive pores that entrap the ground ivy polyphenols, as was also reported by Soliman et al. [31] by increasing only the alginate concentration in the case of cinnamon, clove and thyme oil. The improvement of EE of dandelion polyphenols by reinforcing the alginate with whey protein isolates was reported by Bušić et al. [32] and by Volić et al. [33] for thyme essential oil polyphenols by the incorporation of soy proteins into alginate gel. Among the available literature, only Belščak-Cvitanović et al. [34] have worked on the encapsulation of ground ivy polyphenols. The authors in [34] reported a slightly higher EE (89.37%) than in the present study (84.06%), using a different approach to improve EE: not proteins, but the polysaccharide chitosan incorporated into alginate gel and enhanced with ascorbic acid by electrostatic extrusion.

### 3.3. Physical Characterization of Microparticles

**Figure 1:**
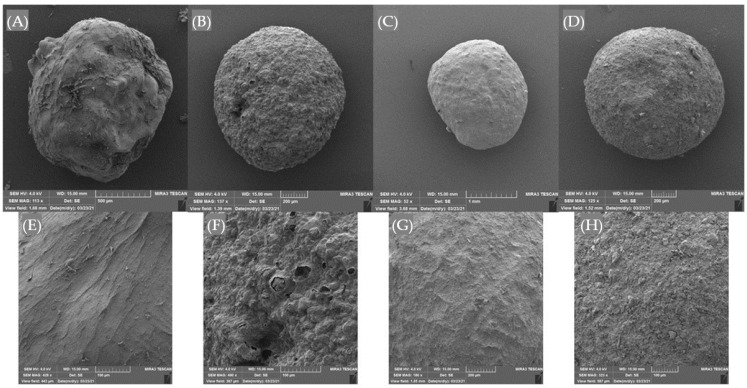
SEM micrographs of (**A**) alginate microparticles (As); (**B**) alginate–rice protein microparticles (A_RICs); (**C**) alginate–pumpkin protein microparticles (A_PUMs); (**D**) alginate–peanut protein microparticles (A_PEAs); surface morphology of (**E**) As; (**F**) A_RICs; (**G**) A_PUMs; (**H**) A_PEAs.

The physical characterization of microparticles included the determination of size and color parameters, and as well as their morphology by applying SEM. The results are presented in Table 3 and Figure 1.

From the presented results, it is evident that all microparticles were uniformed in size, while the A_PUMs were slightly bigger (2.0 mm) than others (1.8 mm). An inversely proportional correlation between size and EE can be noted, since A_PUMs were characterized with the lowest EE for all examined bioactive parameters. In general, microparticles smaller in size are preferred due to later easier application and incorporation in food or other related products. The size of alginate microparticles >1 mm is expected when performing encapsulation by ionic gelation [32,35]. The color of the formulated microparticles was analyzed using CIE L*a*b* color space [36]. According to the presented results (Table 3), the brightest microparticles were the As (L* = 60.66), while the darkest were A_RICs (L* = 40.88). The incorporation of plant proteins into alginate microparticles resulted in the increase in yellow color intensity in all samples, evident by the increase in the b* parameter, and it was most noticeable for A_PUMs (b* = 12.27). A negative value of a* (−0.31) for A_PUMs indicates a green color that is in the accordance with their visual appearance. The highest overall color change, represented by parameter ∆E as a metric for understanding how the human eye perceives color difference, was noted for A_RICs (∆E = 20.92). According to the literature [37], values of ∆E from 11 to 49 denote perception as “colors are more similar than the opposite” and it could be applied to all microparticles. 

The shape and surface morphology of freeze-dried microparticles were examined using SEM, and the results are shown in Figure 1A–H.

Before freeze-drying, all microparticles were mostly spherically and regularly shaped, but after the subjection to freeze-drying, some changes in their morphological characteristics were noted, primarily for alginate microparticles (As). Compared to A_RICs, A_PUMs and A_PEAs, freeze-drying was more destructive for the As, causing undesirable changes in terms of irregular shape and heterogeneous surface (Figure 1A–D). It can be explained by the porous structure of alginate microparticles, causing water leakage during drying, thus destabilizing the gel network and resulting in the collapse of the calcium alginate pores walls and structure cracking [32]. By that claim, the conclusion can be made that the incorporation of plant proteins into alginate gel indeed can strengthen its structure. SEM analysis revelated that the surface of A_RICs, A_PUMs and A_PEAs (Figure 1F–H) was not as smooth as in the As (Figure 1E), but rougher with small protrusions, but it should not be considered a flaw.

### 3.4. Encapsulation Efficiency (EE) and Physical Characterization of Liposomes

The results of liposomal EE and physical characterization are presented in Table 4. 

According to the obtained results, the efficiency of the liposomal encapsulation of TPC (94.66%), as well as the main polyphenolic compound, rosmarinic acid (97.64%), is extremely high. The efficiency of retaining the antioxidant capacity in encapsulated form determined by the ABTS (93.17%) and DPPH (93.26%) methods is also high, reflecting the rich polyphenolic composition of ground ivy extract. In the study of Baranauskaite et al. [38], the liposomal EE of rosmarinic acid extracted from oregano (*Origanum onites* L.) varied between 65%, corresponding to the liposomes prepared only with phosphatidylcholine (Phospholipon 90H), and 90% corresponding to the liposomes consisting of phosphatidylcholine and dimyristoyl phosphatidylglycerol in ratio 1:1. Further, Yücel et al. [39] reported liposomal EE of 62% for rosmarinic acid prepared by the dry film hydration method using dipalmitoyl phosphatidylcholine and cholesterol. Liposomes were also found to be an appropriate encapsulation technique for *Polygonum aviculare* L. extract [40], *Glycyrrhiza glabra* L. roots extract [41], and *Lycium barbarum* L. [42] leaves extract, achieving EE of 83, 84 and 84.6%, respectively.

The physical properties of liposomes that were analyzed were Z-average size (“harmonic intensity averaged particle diameter”), polydispersity index (PDI) and zeta potential. The results are presented in Table 4. The polydispersity index (PDI) is a measure of the width of unimodal size distributions, and acceptable values are below 0.7 [43]. Liposomes loaded with ground ivy extract were more homogenous (PDI = 0.21) and lower in size (106.7 nm) than plain liposomes (PDI = 0.33, size = 192.9 nm). Additionally, both liposomal suspensions were of Z-average size higher than 100 nm, suggesting at least a double-bilayer structure of liposomes [44] and of PDI values more than 0.1, implying polydisperse particle size distributions [45]. Further, due to their charge, liposomes repeal each other with repulsive forces and thus avoid the aggregation and remain separate, making the liposomal suspension stable. A zeta potential higher than 30 mV or less than −30 mV of liposomes is preferable for stable suspension [46]. According to the obtained results for zeta potential, plain liposomes (−27.98 mV) were more stable than loaded liposomes (−21.17 mV).

In a further step, liposomes were coated with alginate–rice protein gel since those microparticles were characterized with the highest EE for all examined bioactive parameters and showed good physical characteristics. 

### 3.5. FT-IR Spectroscopy

**Figure 2:**
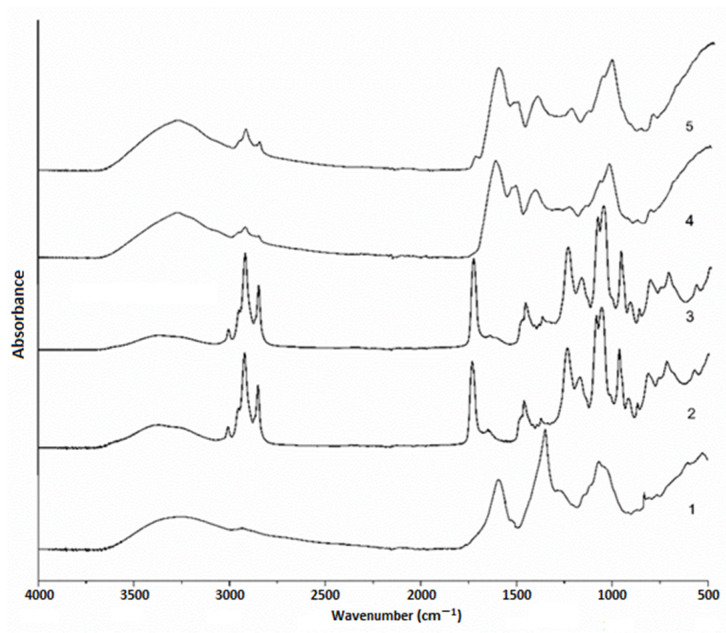
FT-IR spectrum of (1) ground ivy extract; (2) plain liposomes; (3) loaded liposomes; (4) alginate–rice protein microparticles; (5) liposomes covered with alginate–rice protein gel.

FT-IR spectroscopy was applied to investigate the presence of different interactions among ground ivy extract, liposomes, alginate and rice proteins. The FT-IR spectrum of ground ivy extract, plain and loaded liposomes, alginate–rice proteins microparticles and as well as loaded liposomes coated with alginate–rice proteins, are presented in Figure 2.

FT-IR spectrum of the ground ivy extract showed only several prominent non-specific bands. A broad band peaking at 3246 cm^−1^ originates from OH stretching vibrations and it is followed by much less pronounced double bands at 2934 and 2870 cm^−1^ that originate from CH stretching of methyl/methylene groups. A strong band in the carbonyl region of the spectrum, at 1593 cm^−1^, is associated with carboxylate anions, i.e., carboxylic groups, that are present in a vast number of plant secondary metabolites. Sharp band at 1350 cm^−1^ may arise from the OH bending of both alcohol and phenol groups, indicating high content of these functional groups. A group of overlapping bands in the region between 1200 and 800 cm^−1^, peaking at 1070 cm^−1^, may be attributed to various C–O and C–O–C and C–C vibrations of alcohols, acids and sugars.

FT-IR spectrum of plain liposomes showed characteristic bands of phosphatidylcholine. CH and CH2 stretching bands at 2923 and 2853 cm^−1^, arising from fatty acid chains are of much greater intensity when compared to the extract spectrum. A small and sharp band at 3009 cm^−1^ is attributed to the CH stretching of *N*-CH3 groups. Another characteristic band at 1735 cm^−1^ corresponds to carbonyl group (C = O) of ester bond between glycerol and fatty acids. Bands at 1239 and 1088 cm^−1^ arise due to anti-symmetric and symmetric stretching of PO2-, respectively, while the band at 1060 cm^−1^ may be associated with C–O stretching vibrations. The quaternary ammonium group of choline moiety gives rise to two bands, at 968 and 921 cm^−1^, which correspond to the anti-symmetric and symmetric stretching of the C–N bond, respectively. Although FT-IR analysis was performed immediately after the freeze-drying process, there is a visible presence of water in the sample. A broad band at 3368 cm^−1^ (OH stretching) is accompanied by a smaller, symmetrical OH scissors band at 1648 cm^−1^, implying that there is chemically bound water on the liposome surface, probably interacting with phosphate groups through hydrogen bonds.

The FT-IR spectrum of the liposomes loaded with the ground ivy extract mostly resembled that of plain liposomes, which may be due to a relatively low concentration of the extract, but also due to the effect of “shielding” [47] as the extract compounds are water soluble and are expected to be completely enveloped by phospholipid layers. PO2- anti-symmetric stretching band (at 1238 cm^−1^) is marked as being particularly sensitive to any structural changes of phosphatidylcholine vesicles, which is more pronounced in the hydrated state [48]. Frías et al. [49] found that arbutin, glycosylated hydroquinone found in bearberry, affected both of the PO2- stretching bands’ frequency depending on its positioning within the multi-layered phosphatidylcholine micelles. In the same study [49], the band associated with symmetric PO2- stretching (1088 cm^−1^) was affected only if arbutin was outside, while the antisymmetric stretching band (1238 cm^−1^) was affected by arbutin being both inside and outside, leading to their downshift. Phenolic compounds in the present study of the ground ivy extract may act in the same way, causing a slight shift of the PO2- anti-symmetric stretching band. This shift can be observed in the spectra of plain and loaded liposomes, with band’s peak at 1238 cm^−1^ in the former moving to 1243 cm^−1^ in the latter. The other visible change in the FT-IR spectrum of the loaded liposomes is the presence of a low-intensity band associated with carboxylic groups (at ~1600 cm^−1^), clearly originating from the extract compounds, indicating that some compounds with carboxylic groups may be “entrapped” on the surface. Further evidence that some of the extract compounds are incorporated at the surface level of liposomes is the diminishing of the intensity of the bands associated with bound water (at 3368 and 1648 cm^−1^). Compounds that are capable of interacting through hydrogen bonds may replace water molecules adsorbed on the liposome surface. Due to the extract’s complexity and its relatively low concentration, it is impossible to completely understand the liposome structure, but evidence that comes from the FT-IR analysis suggests that the extract compounds are not only entrapped within the liposomes, but also on their surface, having an impact on their physico-chemical characteristics.

Furter, in the FT-IR spectrum of alginate microparticles, a band at 1600 cm^−1^ is dominating, representing the asymmetric stretching of carbonyl (C=O) bond of the carboxylate anion. Several overlapping bands between 1200 and 800 cm^−1^ (“fingerprint region”) represent various C–O, C–O–C and C–C vibrations. In the FT-IR spectrum of alginate–rice protein microparticles, on the other hand, the most pronounced bands are amide I (~1630 cm^−1^) and amide II band (1550 cm^−1^). Alginate–rice protein microparticles show the coupling of the carboxylate and amide I band, resulting in a new band peaking at 1623 cm^−1^, as well as the coupling of bands in the fingerprint region. The addition of liposomes showed little changes in the FT-IR spectrum. A phosphatidylcholine ester band at 1723 cm^−1^ became visible, as well as PO2- antisymmetrical stretching band (at 1240 cm^−1^) and C–N antisymmetrical stretching band (968 cm^−1^) as a shoulder on the much broader band at 1053 cm^−1^.

### 3.6. Bioactive Characterization of Formulated Agar-Agar Candies

The bioactive characterization of formulated candies without (CAN_1) and with incorporated liposomes coated with alginate–rice proteins (CAN_2) was performed; the results are presented in Table 5.

From the obtained results, it is evident that the incorporation of ground ivy extract in both candy formulations resulted in enrichment with phenolic compounds. In the sample CAN_2, liposomal encapsulated systems coated with alginate–rice protein gel additionally contributed to the enhancement of bioactive potential by increasing TPC, DPPH-, ABTS-measured antioxidant capacity and rosmarinic acid content by 10.98, 10.21, 10.13 and 6.26%, respectively, compared to the sample CAN_1. Additionally, formulated candies were sensory evaluated with high scores, regarding the overall acceptance (data not shown).

### 3.7. Simulated Gastro-Intestinal Digestion

**Figure 3:**
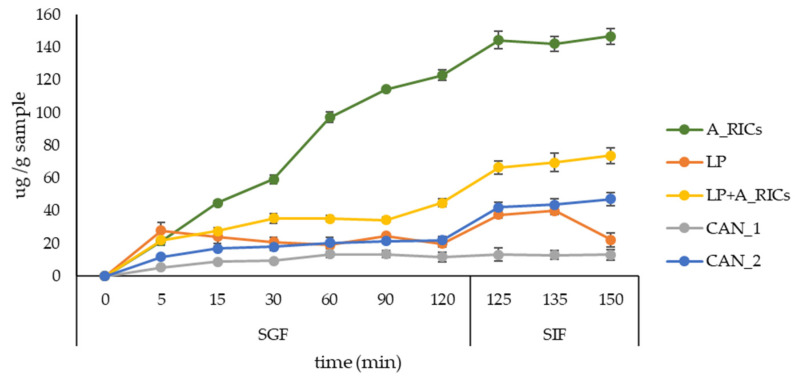
Release of rosmarinic acid in simulated gastro-intestinal digestion from alginate–rice proteins microparticles (A_RICs), loaded liposomes (LP), loaded liposomes coated with alginate–rice proteins gel (LP + A_RICs), candies with incorporated ground ivy extract (CAN_1) and candies with incorporated ground ivy extract and LP + A_RICs (CAN_2).

The release of rosmarinic acid from encapsulated systems and formulated candies was performed simulating digestion in the stomach (SGF) and small intestine (SIF). The results are presented in Figure 3.

All encapsulation systems showed the gradual release of rosmarinic acid during 120 min in SGF, followed by continued release in SIF up to 30 min. Regarding the alginate–rice proteins microparticles, the higher release of rosmarinic acid occurred when they were placed in SIF, as was expected because alginate microparticles are characterized as stable in acidic solutions and degrade under higher pH value [50]. However, the weaking of the alginate hydrogel network appears because of the displacement of Ca^2+^ ion moieties by the monovalent cations present in SGF [51]. Higher release of rosmarinic acid from liposomes was also observed in SIF. It was reported previously that low pH and the presence of pepsin in SGF have little effect on liposomes [52], while bile salts and pancreatin in SIF affects their physical characteristics and morphology due to the hydrolysis of phospholipids by pancreatin enzymes and the interaction of bile salts with the components of liposome [53]. The detergent property of bile salts can disrupt the phospholipid bilayers in the liposomes [53]. Similar was observed in other study [54], where the integrity of the liposomes remained unchanged when digested in SGF, while a damaged membrane and the release of free fatty acids, facilitated by the presence of bile salts, appeared in SIF. Further, liposomes achieved a maximum release of rosmarinic acid at 15 min in SIF, while in coated form with alginate–rice proteins the controlled release was prolonged up to 30 min. Although candies prepared only with ground ivy extract (CAN_1) exhibited potent bioactive potential (Section 3.6.), they did not provide a gradual release of rosmarinic acid during simulated gastro-intestinal digestion, while those prepared with encapsulation system (CAN_2) enabled the desirable simulated digestion of rosmarinic acid.

## 4. Conclusions

Ground ivy (*Glechoma hederacea* L.) has shown to be a valuable source of natural bio-active compounds, especially rosmarinic acid. Liposomal encapsulation provided the high retention of rosmarinic acid, but to achieve a more desirable release profile of rosmarinic acid in gastro-intestinal digestion, liposomes should be coated with some biopolymers. In the present study, combination of alginate and plant proteins, showed to be an excellent choice. The controlled and continuous release of rosmarinic acid from encapsulation systems was retained when incorporated into candies. Therefore, they can serve as a delivery system of bioactive compounds for human health to many people since they are popular confectioneries among all generations. However, future studies should focus on the acceptance of such products by the public and whether they will be accepted by a larger number of consumers.

## Figures and Tables

**Table 1:** Bioactive characterization of ground ivy extract.

TPC (mg GAE/L)	Antioxidant Capacity				Rosmarinic Acid (mg/L)
	DPPH (mmol TroloxE/L)	ABTS (mmol TroloxE/L)	MCC (%)	β-CB (%)	
1186.20 ± 12.75	3.33 ± 0.01	4.05 ± 0.01	74.35 ± 2.26	57.37 ± 6.88	46.04 ± 0.15

TPC-total phenolic content; GAE-gallic acid equivalents; TroloxE-Trolox equivalents; MCC-metal chelating capacity; β-CB- inhibition of β-carotene bleaching.

**Table 2:** Encapsulation efficiency (%) of formulated microparticles.

Sample	TPC	Antioxidant Capacity		Rosmarinic Acid
		DPPH	ABTS	
As	75.15 ± 0.02	70.41 ± 0.03	72.48 ± 0.08	62.44 ± 0.10 ^ab^
A_RICs	84.06 ± 0.05	79.09 ± 0.03	81.10 ± 0.06	78.16 ± 0.01
A_PUMs	65.20 ± 0.07	71.96 ± 0.08	74.82 ± 0.03	63.54 ± 0.03 ^ac^
A_PEAs	62.12 ± 0.02	67.23 ± 0.01	71.65 ± 0.05	61.57 ± 4.34 ^bc^

As = alginate microparticles; A_RICs = alginate–rice protein microparticles; A_PUMs = alginate–pumpkin protein microparticles; A_PEAs = alginate–peanut protein microparticles; TPC = total phenolic content; Means in the same column denoted with the same superscript letters are not significantly different (*p* > 0.05).

**Table 3:** Visual appearance, size and color parameters (L*, a* and b*) of microparticles.

Sample	Visual Appearance of Microparticles	Size (mm)	L*	a*	b*	∆E
As	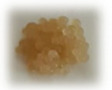	1.8 ± 0.0 ^ab^ mm	60.66 ± 0.96	−0.15 ± 0.08	5.17 ± 0.86	/
A_RICs	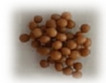	1.8 ± 0.0 ^ac^ mm	40.88 ± 0.15	3.65 ± 0.11	10.74 ± 0.93	20.92 ± 0.24
A_PUMs	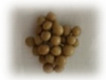	2.0 ± 0.0 mm	51.37 ± 0.80	−0.31 ± 0.08	12.27 ± 0.58	11.74 ± 0.32
A_PEAs	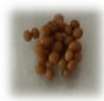	1.8 ± 0.0 ^bc^ mm	46.24 ± 0.40	2.56 ± 0.09	7.87 ± 0.19	14.92 ± 0.34

As = alginate microparticles; A_RICs = alginate–rice proteins microparticles; A_PUMs = alginate–pumpkin proteins microparticles; A_PEAs = alginate–peanut proteins microparticles; Means in the same column denoted with the same superscript letters are not significantly different (*p* > 0.05).

**Table 4:** Encapsulation efficiency (EE) and physical characterization of liposomes.

	EE (%)				Physical Characterization		
Sample	TPC	Antioxidant Capacity		Rosmarinic Acid	Z-Average Size (nm)	PDI	Zeta Potential (mV)
		DPPH	ABTS				
Plain liposomes	/				192.9 ± 4.6	0.33 ± 0.01	−27.98 ± 0.98
Loaded liposomes	94.66 ± 0.38	93.26 ± 1.20	93.17 ± 0.58	97.64 ± 0.25	106.7 ± 0.9	0.21 ± 0.01	−21.17 ± 0.46

TPC = total phenolic content; PDI = polydispersity index.

**Table 5:** Bioactive characterization of formulated candies.

Sample	TPC (mg GAE/g)	Antioxidant Capacity (µmol TroloxE/g)		Rosmarinic Acid (µg/g)
		DPPH	ABTS	
CAN_1	0.73 ± 0.01	4.13 ± 0.02	4.79 ± 0.04	38.93 ± 0.48
CAN_2	0.82 ± 0.00	4.60 ± 0.00	5.33 ± 0.02	41.53 ± 0.77

TPC, total phenolic content; GAE, gallic acid equivalents; TroloxE, Trolox equivalents. Values for TPC, DPPH-, ABTS-measured antioxidant capacity and rosmarinic acid content between CAN_1 and CAN_2 are significantly (*p* < 0.05) different, determined by single-factor ANOVA.

## Data Availability

Data are contained within the article.

## Supplementary Materials

The following supporting information can be downloaded at: https://www.mdpi.com/article/10.3390/foods11121816/s1, Figure S1: HPLC chromatogram (recorded at 320 nm) of investigated ground ivy extract, Table S1: Retention time and wavelength (λ) of detection of rosmarinic acid.
