# Supplementary material for: Development, Characterization and Incorporation of Alginate-Plant Protein Covered Liposomes Containing Ground Ivy (Glechoma hederacea L.) Extract into Candies
Source: Foods. 2022 Jun 20;11(12):1816. doi: 10.3390/foods11121816 (PMC9222263; doi:10.3390/foods11121816)
Supplement: Supplementary file 1 [file foods-11-01816-s001.zip › foods-1692884-supplementary.pdf]

Supplementary Materials

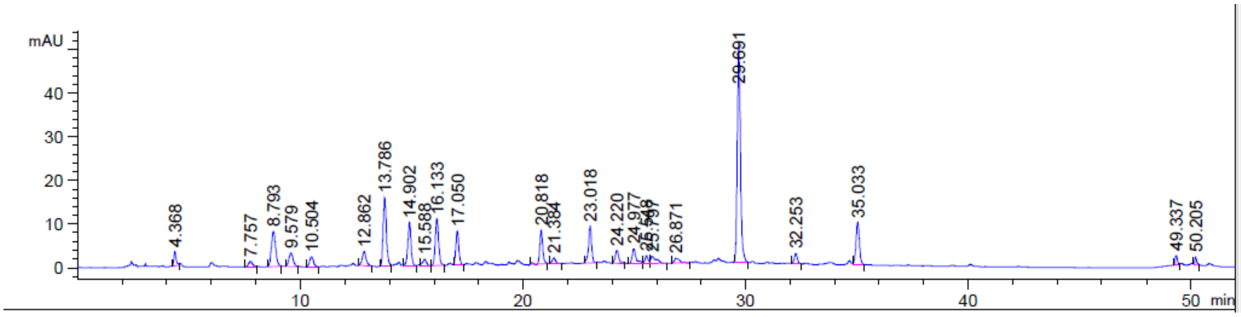

Figure S1. HPLC chromatogram (recorded at 320 nm) of investigated ground ivy extract

Table S1. Retention time and wavelength (λ) of detection of rosmarinic acid

Phenolic compound	Retention time	λmax
Rosmarinic acid	29.6 min	320 nm
